# Novel Axillary Approach for Brachial Plexus in Robotic Surgery: A Cadaveric Experiment

**DOI:** 10.1155/2014/927456

**Published:** 2014-07-21

**Authors:** Cihangir Tetik, Metin Uzun

**Affiliations:** ^1^Department of Hand Surgery, Acıbadem Maslak Hospital, Darüşşafaka Mah. Büyükdere Cad. No. 40, Maslak, Sarıyer, Istanbul, Turkey; ^2^Orthopaedics and Traumatology Department, Acıbadem Maslak Hospital, Darüşşafaka Mah. Büyükdere Cad. No. 40, Maslak, Sarıyer, Istanbul, Turkey

## Abstract

Brachial plexus surgery using the da Vinci surgical robot is a new procedure. Although the supraclavicular approach is a well known described and used procedure for robotic surgery, axillary approach was unknown for brachial plexus surgery. A cadaveric study was planned to evaluate the robotic axillary approach for brachial plexus surgery. Our results showed that robotic surgery is a very useful method and should be used routinely for brachial plexus surgery and particularly for thoracic outlet syndrome. However, we emphasize that new instruments should be designed and further studies are needed to evaluate in vivo results.

## 1. Introduction

Brachial plexus surgery using the da Vinci surgical robot is a new procedure [1].

To evaluate the advantages and the restrictions of the technique, a cadaveric study of supraclavicular and axillary approaches was conducted. We found that the axillary approach was useful and advantageous for lower roots, particularly for thoracic outlet syndrome (TOS). This report will focus on the evaluation of axillary robotic approach as the advantages and disadvantages of supraclavicular robotic intervention have been widely discussed in the literature.

### 1.1. Surgical Procedure

**Figure 1:**
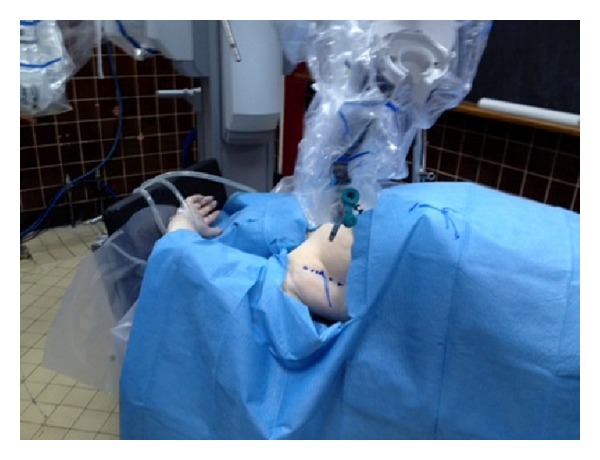
The picture showing setup position of the cadaver.

**Figure 2:**
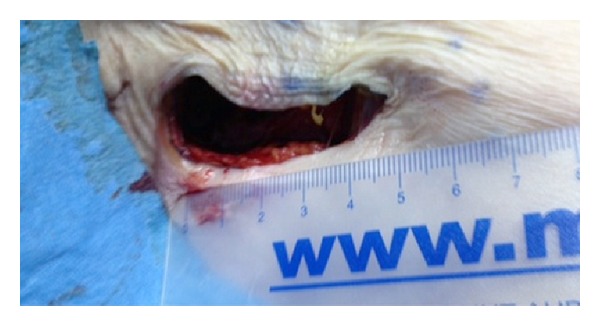
The picture showing the incision.

**Figure 3:**
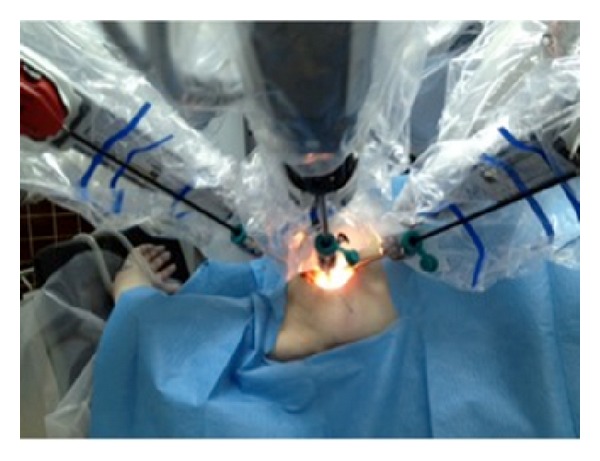
The picture showing the setup position of the robot.

**Figure 4:**
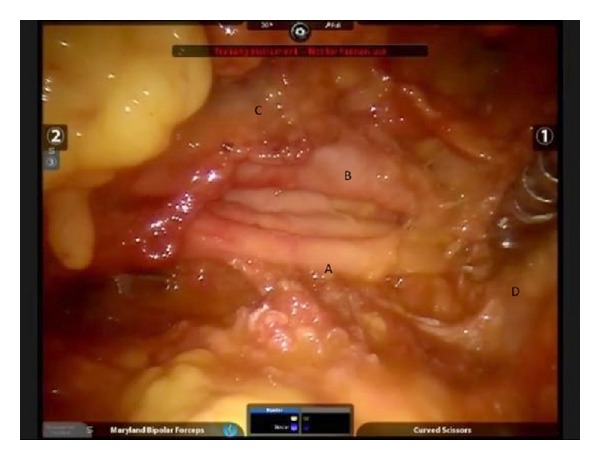
The picture showing the anatomical exposure of the lower part of the brachial plexus. A-lower truncal level, B-subclavian artery, C-subclavian vein, and D-fist rib.

A human cadaver was subjected to this experiment in Paris University Ecole Europèenne de Chirurgie anatomy laboratory and da Vinci robot system was used. The cadaver was placed supine on the operating table. The left arm was tucked along the side and the right arm was placed in a semiflexed position extending toward the anesthesia location near the head, supported by foam and blankets (Figure 1). A 6 cm long incision was made at the right axillar line, lateral to the edge of the pectoralis major muscle (Figure 2). Blunt dissection was performed to create the working space area. A self-retaining Chung retractor was placed into the incision to elevate the pectoralis major muscle flap. The robot was docked as a camera; right and left robotic arm were adapted in the incision area (Figure 3). A 10 mm 0^0^ downlooking scope, Maryland forceps, and a curved scissors were introduced through the incision. The working space was maintained with the self-retaining retractor, without CO_2_ insufflation (Figure 4). First rib was found; C8-T1 and lower truncus were identified.

The subclavian artery was seen in front of the truncus and was positioned to the posterior of the working space. Anterior scalene muscle attachment and subclavian vein were seen anterior to the muscle. Subclavian artery was dissected from the plexus and truncus of the lower plexus was exposed with blunt dissection. The plexus was exposed thoroughly from T1 to C7 levels. In this surgical setting, the operating surgeon, who has a wide experience in open brachial surgery of the brachial plexus, reported that lower brachial plexus exposure was easier from the axillary working area and a more wide range of motion was achieved to manipulate the robotic tools compared to the supraclavicular exposure for lower part of the brachial plexus.

## 2. Discussion

The development of robotic-assisted minimally invasive techniques began in urology, general surgery, and gynecology because of the generally large working spaces available in the abdomen for these types of surgeries [1–4]. Since then, other surgeons have sought to use robotic devices in other areas, such as the brachial plexus [5, 6].

Brachial plexus dysfunction can be the result of shoulder trauma [7, 8]. Palsy may occur with shoulder dislocation and/or traction injuries. It can also occur with TOS, which encompasses three separate disorders involving compression of the subclavian artery, subclavian vein, or brachial plexus in the triangular space bordered by the first rib, clavicle, and scalene muscles [9, 10]. Compression of the vessel-nerve package at the thoracic inlet has been treated with soft-tissue (scalene muscle) release and/or bone (first rib) resection [9]. Surgical approaches to first rib resection may be transthoracic, transaxillary, supraclavicular, infraclavicular, or thoracoscopic [9, 10]. However, these approaches are typically associated with incomplete resection of the most medial portion of the first rib and neurovascular complications [11]. Theoretically, a minimally invasive transthoracic approach can obviate these problems, enabling complete resection of the offending portion of the first rib without neurovascular complication. Gharagozloo et al. and Martinez et al., respectively, reported successful results of robotic en bloc first rib resection for TOS treatment via transthoracic and transaxillary approaches [9, 11]. Gharagozloo et al. and Martinez et al.'s techniques were only bony interventions and as being intrathoracic these need to be lung collapsed and lung complication can be waiting risk.

Open brachial plexus interventions can be performed using a supraclavicular or axillary approach. Although Liverneaux et al. reported techniques and results of upper brachial plexus injury intervention* via* robotic surgery with a supraclavicular approach, they described the disadvantages as a narrow working space and difficulty to expose the C7 vertebra [1, 5, 12]. To our knowledge, this report is the first to objectively describe robotic axillar brachial plexus exposure. Thus, we discuss the theoretical and clinical advantages and disadvantages of the axillary approach in the present report.

### 2.1. Benefits of Robotic Surgery

The development of robot-assisted surgery has revealed new perspectives in peripheral nerve microsurgery. Minimally invasive robot-assisted surgery could lead to modification of the classic algorithm for the treatment of traumatic brachial plexus lesions [6, 8]. To date, exploration of these lesions has not been attempted less than 3 months after the traumatic event because clinical examination cannot provide an accurate diagnosis or reliable prognosis in these first weeks [13]. Early intervention may enable initial assessment of the lesion and repair of potentially graftable nerve roots. Several robotics properties are particularly adapted to microsurgery, such as high-resolution three-dimensional (3D) visualization with up to ×40 magnification, up to 10-fold magnification of surgical movements, elimination of physiological tremors, and the provision of ergonomic work conditions for otherwise uncomfortable surgery. Robotic surgical systems allow high-definition magnified 3D visualization of the operative field, provide significant instrument maneuverability, even within a confined space, and may overcome the shortcomings of conventional approaches [2, 5].

Axillary (infraclavicular) brachial plexus intervention* via* robotic surgery has not been described previously. Axillary intervention was previously performed as an open procedure to expose the plexus or resect the first rib for the treatment of TOS [9]. Martinez et al. described first rib resection* via* robotic surgery but not to address plexus injury without transthoracic exposure, a novel minimally invasive approach to the first rib from inside of the chest [9]. In addition, Gharagozloo et al. reported first rib resection via transthoracic robotic surgery for Paget-Schroetter disease [11]. Martinez et al.'s techniques were considered more useful for lower brachial plexus viewing and assessing according to Gharagozloo et al.'s but these two techniques describe only bony interventions.

### 2.2. Disadvantages of Robotic Surgery

The experience of the whole surgical team with robotic technology is important for the procedure. During learning curve period, two staff surgeons are required to participate in all procedures to ensure the safety of the program [5, 9]. Martinez et al. reported importance of the learning curve, not only for the surgeon but also for the entire surgical team and 180 minutes for the initial 10 cases [9].

A second problem associated with robotic surgery is patient selection [1]. Reported exclusion criteria include a history of previous incision in the same area and obesity, which present difficulties in robotic surgery initiation. We agree with these criteria.

Other drawbacks of this new surgical approach are the increased cost of surgical equipment and longer operating time, especially during the learning curve period. However, we believe that the avoidance of a classic incision leads to significant patient satisfaction for cosmetic reasons and we believe that demand for this procedure from a select group of patients justifies the exploration of alternative ways to avoid classic brachial plexus exposure.

## 3. Conclusion

This report presents our initial experience with robot-assisted axillary exposure of the brachial plexus region. In our opinion robotic surgery will be used routinely in the future for brachial plexus surgery and particularly for TOS that is caused by bone and/or soft tissue. However, newer dedicated surgical instruments need to be developed and further studies should be conducted to evaluate in vivo application and results of this novel approach.

## Supplementary Material

This video show axillary approach to the brachial plexus. da Vinci robot is set through the axillary insicion caudal-cranial direction. Lower part of the plexus C7,C8 and T1 are dissected with specific instruments. Subclavian artery and vein is dissected easily and scalenius anterior muscle is released.
